# *WAKL8* Regulates Arabidopsis Stem Secondary Wall Development

**DOI:** 10.3390/plants11172297

**Published:** 2022-09-02

**Authors:** Yingxuan Ma, Luke Stafford, Julian Ratcliffe, Antony Bacic, Kim L. Johnson

**Affiliations:** 1 School of BioSciences, University of Melbourne, Parkville, VIC 3052, Australia; 2 La Trobe Institute for Agriculture & Food, Department of Animal, Plant and Soil Science, AgriBio Building, La Trobe University, Bundoora, VIC 3086, Australia; 3 Sino-Australia Plant Cell Wall Research Centre, College of Forestry and Biotechnology, Zhejiang Agriculture and Forestry University, Lin’an, Hangzhou 311300, China

**Keywords:** wall-associated kinases (WAKs)/kinase-likes (WAKLs), secondary cell wall (SCW), cell wall integrity (CWI), cellulose, lignin

## Abstract

Wall-associated kinases/kinase-likes (WAKs/WAKLs) are plant cell surface sensors. A variety of studies have revealed the important functions of WAKs/WAKLs in regulating cell expansion and defense in cells with primary cell walls. Less is known about their roles during the development of the secondary cell walls (SCWs) that are present in xylem vessel (XV) and interfascicular fiber (IF) cells. In this study, we used RNA-seq data to screen *Arabidopsis thaliana* WAKs/WAKLs members that may be involved in SCW development and identified *WAKL8* as a candidate. We obtained T-DNA insertion mutants *wakl8-1* (inserted at the promoter region) and *wakl8-2* (inserted at the first exon) and compared the phenotypes to wild-type (WT) plants. Decreased *WAKL8* transcript levels in stems were found in the *wakl8-2* mutant plants, and the phenotypes observed included reduced stem length and thinner walls in XV and IFs compared with those in the WT plants. Cell wall analysis showed no significant changes in the crystalline cellulose or lignin content in mutant stems compared with those in the WT. We found that *WAKL8* had alternative spliced versions predicted to have only extracellular regions, which may interfere with the function of the full-length version of *WAKL8*. Our results suggest WAKL8 can regulate SCW thickening in Arabidopsis stems.

## 1. Introduction

Plant cell walls are functional dynamic networks that can both maintain integrity and react to intra- and extracellular stimuli [1,2,3]. Plant cells have complex and precise cell wall sensing networks that facilitate the cell wall modifications appropriate for growth and in response to environmental conditions. Numerous cell wall integrity (CWI) sensors have been proposed, and include arabinogalactan-proteins (AGPs), glycosylphosphatidylinositol (GPI)-anchored proteins (GPI-APs), DEFECTIVE KERNEL1 (DEK1), receptor-like kinase (RLK) family members including *Catharanthus roseus* receptor-like kinase (CrRLK1L), leucine-rich repeat receptor kinases (LRR-RLKs), L-lectin RLKs, plant external response-like kinases (PERKs), lysine-motif containing receptor-like kinases (LysM-RLKs), and wall-associated kinases/kinase-likes (WAKs/WAKLs) [1,4,5,6,7,8,9,10,11,12]. In general, RLKs, mechanosensors/channels, and glycoproteins are the major categories of CWI sensors that can intersect with other wall regulatory pathways, including hormones and reactive oxygen species (ROS).

One of the most well-characterized CWI sensors is the CrRLK1L kinase FERONIA, which interacts with extracellular signal peptides and RALFs, and can regulate cell growth, female gametophyte development, and defense via downstream signaling pathways that intersect with ROS production, calcium, and mitogen-activated protein kinases (MPKs) [13]. Other RLKs proposed to be CWI sensors include THE1, shown to regulate cellulose-deficiency-induced stress responses, and WAKs/WAKLs that can interact with pectin/pectin fragments and regulate cell growth and defense [9,13]. WAK/WAKL family members share common structures of an N-terminal carbohydrate binding domain (CBD), epidermal growth factor 2-like (EGF2-like) domain, calcium binding EGF (EGF-Ca^2+^) domain, transmembrane domain (TMD), and intracellular Ser/Thr protein kinase sites (PK) [14,15]. Members of the WAKs/WAKLs family have been shown to be covalently linked with either cell wall pectin or pectic fragments and to trigger downstream signaling pathways involving different MPKs [9,14,16,17,18,19]. The WAKs/WAKLs family has been shown to play important functions in regulating plant growth, development, and resistance to pathogens in a variety of plant species, including Arabidopsis, maize, rice, and cotton, by associating with primary cell walls [20,21,22,23,24,25,26,27,28,29,30,31]. However, the role of WAKs in regulating SCW development is still poorly understood.

The secondary cell wall (SCW) of the stem xylem vessel (XV) and the interfascicular fiber (IF) cells constitute the major components of renewable resources and are important structures for plant growth, development, and response to stresses [32]. Cellulose, xylan, and lignin are major polymers of SCWs, with (glycol) proteins as a minor component [32]. The initiation and development of SCW are regulated by transcription factor hierarchies [33]. In addition, environmental stimuli such as blue light, cold stress, and mechanical stress have also been suggested to integrate with transcription factors to regulate SCW development [34,35,36,37]. Fasciclin-like arabinogalactan-protein 11 (FLA11) was shown to be SCW-specific, and overexpression FLA11 (OE-FLA11) showed the early initiation and altered composition of SCWs, leading to proposed roles as a CWI sensor involved in sensing mechanical stimuli [36]. However, the role(s) of CWI sensors regulating SCW development is still poorly understood. Recently, functions for WAKs/WAKLs in regulating SCW development have been suggested. The expression of a rice *WAK*, *Xa4*, is predominantly in stem sclerenchyma cells and tightly correlates with SCW cellulose synthesis genes [38]. A large family of *WAKs* with 175 members in the model tree species, *Populus*, used to study wood formation, was also identified [39].

In this study, we screened the gene expression levels of *WAKs/WAKLs* in Arabidopsis stems and identified *WAKL8* as a putative candidate in regulating stem SCW development. We obtained and phenotypically analyzed T-DNA insertion mutants of *wakl8-1* and *wakl8-2* and found that WAKL8 can regulate stem development, XV, and IF wall thickening. We identified an alternative spliced version of *WAKL8* predicted to encode a protein lacking the EGF-Ca^2+^, transmembrane, and intracellular domains and proposed functions for this variant.

## 2. Results

### 2.1. Identification of WAK/WAKL Family Genes during Stem Secondary Wall Development

**Figure 1:**
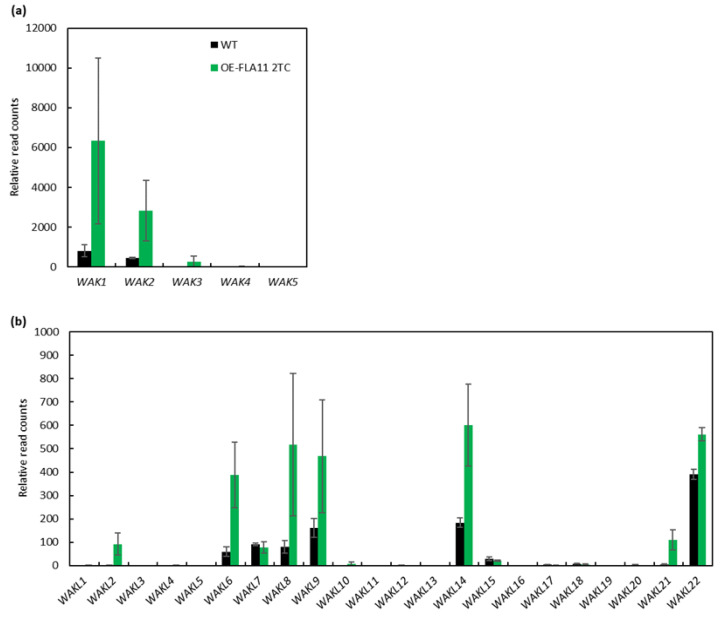
Comparison of *WAKs/WAKLs* transcript levels in OE-FLA11 and WT stems. Analysis of differentially expressed genes (DEGs) in OE-FLA11 stems compared with WT plants revealed upregulation of *WAKs* (**a**) and *WAKLs* (**b**) *WAK1*, *WAK2*, *WAK3*, *WAKL2*, *WAKL6*, *WAKL8*, *WAKL9*, *WAKL14*, *WAKL21*, and *WAKL22*, as shown by the relative read counts from RNA-seq.

**Figure 2:**
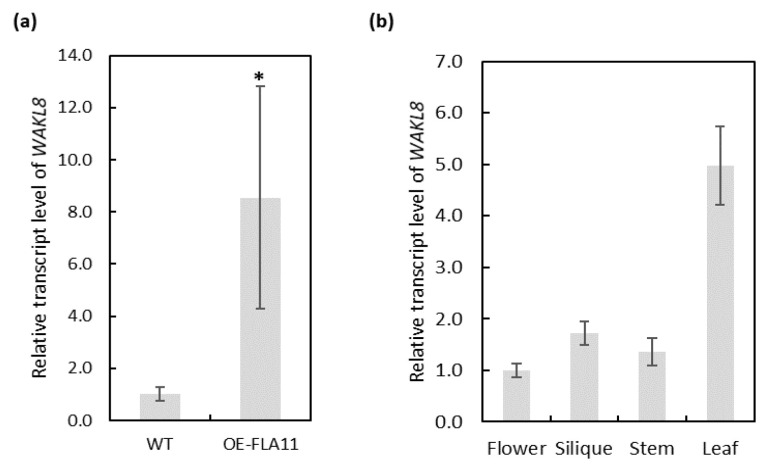
Q-PCR analysis of *WAKL8* transcript levels in OE-FLA11 stems and expression profile in WT tissues. (**a**) Q-PCR analysis showed upregulation of *WAKL8* transcript in OE-FLA11 stems compared with that in WT stems. (**b**) Q-PCR analysis showed *WAKL8* is ubiquitously expressed in different tissues but highest in leaves. Data shown as average ± SD. *n* ≥ 3 plants acquired from 3 biological replicates. * Significant difference compared with WT plants, *p* < 0.05 using Student’s *t*-test.

The *WAK/WAKL* family in Arabidopsis consists of more than 27 members [40]. To identify which members are potentially involved in SCW development, we used a combination of expression levels of *WAKs/WAKLs* in the RNA-seq data of OE-FLA11 plant stems that showed earlier onset of SCW development than WT and Arabidopsis eFP browser data to narrow the targets [36,41,42,43]. Ten *WAKs/WAKLs* were found to have altered transcript levels in OE-FLA11 young stems compared with WT plants: *WAK1*, *WAK2*, *WAK3*, *WAKL2*, *WAKL6*, *WAKL8*, *WAKL9*, *WAKL14*, *WAKL21*, and *WAKL22* (Figure 1a,b). The RNA-seq data showed *WAKs/WAKLs* with more than 200 read counts and at least two-fold changes in OE-FLA11 compared with WT plants: *WAK1*, *WAK2*, *WAK3*, *WAKL6*, *WAKL8*, *WAKL9,* and *WAKL14* (Figure 1a,b). *WAKL8* (AT1G16260) was recently identified to be expressed at vascular tissues and to play a role in regulating leaf phloem sucrose loading via phosphorylating sucrose transporter 2 (SUC2) [44]. Q-PCR analysis was performed to check *WAKL8* expression levels in OE-FLA11 stems compared with WT stems and showed consistent results with those of RNA-seq (Figure 2a). Comparison of *WAKL8* expression levels between the flower, silique, stem, and leaf showed *WAKL8* was broadly expressed in all tissues but higher in the leaves (Figure 2b). The eFP browser visualization of *WAKL8* expression in primary root showed higher levels in vasculatures than in other cell types (Figure S1).

### 2.2. WAKL8 Can Regulate Plant Stem Growth

**Figure 3:**
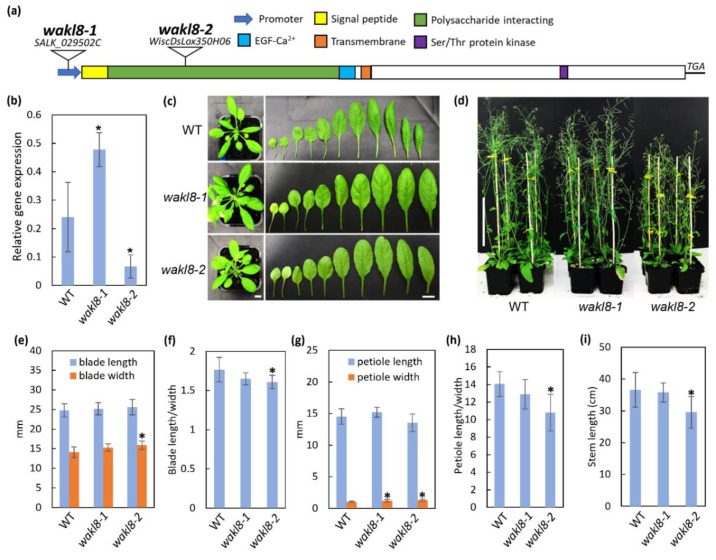
Identification of T-DNA insertions in *WAKL8* and analysis of phenotypes of *wakl8* mutant plants compared with WT plants. (**a**) Schematic of the *WAKL8* coding sequence showing the predicted protein domains and position of the T-DNA inserts for both *wakl8-1* and *wakl8-2*. The *WAKL8* transcript is predicted to encode an N-terminal signal peptide (yellow), followed by a putative extracellular polysaccharide-interacting domain (green), epidermal growth factor-Calcium interacting domain (EGF-Ca^2+^) (blue), single transmembrane domain (orange), and cytoplasmic Ser/Thr protein kinase domain (purple). The T-DNA in the *wakl8-1* mutant is inserted in the promoter region, and for the *wakl8-2* mutant, the T-DNA is inserted in the first exon. (**b**) Q-PCR analysis of *WAKL8*. Expression levels are relative to *ACT2* and show slightly increased *WAKL8* transcript levels in *wakl8-1* plants and decreased *WAKL8* transcript levels in *wakl8-2* plants compared with WT plants. Data shown as average ± SD. *n* ≥ 3 plants. (**c**) Representative image of WT, *wakl8-1*, and *wakl8-2* mutant plants at stage 6.1 [45]. Scales: 1 cm. (**d**) Representative image of WT, *wakl8-1*, and *wakl8-2* mutant plants at stage 6.9 [45]. Scales: 10 cm. (**e–h**) Quantification of leaf blade and petiole length and width of WT, *wakl8-1*, and *wakl8-2* mutant plants at stage 6.1. (**i**) Quantification of stem length of WT, *wakl8-1*, and *wakl8-2* mutant plants at stage 6.9. Stem length is significantly reduced in *wakl8-2* mutant plants compared with that in WT plants. Data shown as average ± SD. *n* ≥ 6 plants. * Significant difference compared with WT plants, *p* < 0.05 using Student’s *t*-test.

*WAKL8* transcripts predict an *N*-terminal signal peptide followed by an extracellular polysaccharide-interacting domain (ECD), EGF-Ca^2+^ domain, single transmembrane domain, and intracellular Ser/Thr kinase domain (Figure 3a). T-DNA insertion mutants in *WAKL8* were obtained for phenotypic analysis. The *wakl8-1* and *wakl8-2* mutants had an insertion in the promoter region and in the first exon, respectively (Figure 3a). The *WAKL8* transcript level was slightly upregulated in *wakl8-1* stems compared with that in WT stems and decreased to about 20% of the WT levels in the *wakl8-2* mutant (Figure 3b). Observations of plant growth showed that *wakl8-1* and *wakl8-2* plants had different rosette leaf shapes compared with the WT plants (Figure 3c), and mature *wakl8-2* plants were shorter than WT and *wakl8-1* plants (Figure 3d). Measurements of blade length and width showed *wakl8-2* plants had a higher blade width and reduced length/width ratio than WT and *wakl8-1* plants (Figure 3e,f). Measurements of petiole length and width showed *wakl8-1* and *wakl8-2* plants had a higher petiole width than WT plants (Figure 3g), and *wakl8-2* plants had a reduced length/width ratio compared with WT and *wakl8-1* plants (Figure 3h). Changes in SCWs are often revealed by defects in stem development. Measurements of stem length of stage 6.9 plants [45] were also conducted, as this is where we observed that SCW defects occurred and found that *wakl8-1* plants had a similar stem length as WT plants, whereas *wakl8-2* plants had a significantly reduced stem length (Figure 3i).

### 2.3. Histological Analyses of wakl8-1 and wakl8-2 Mutant Stems

**Figure 4:**
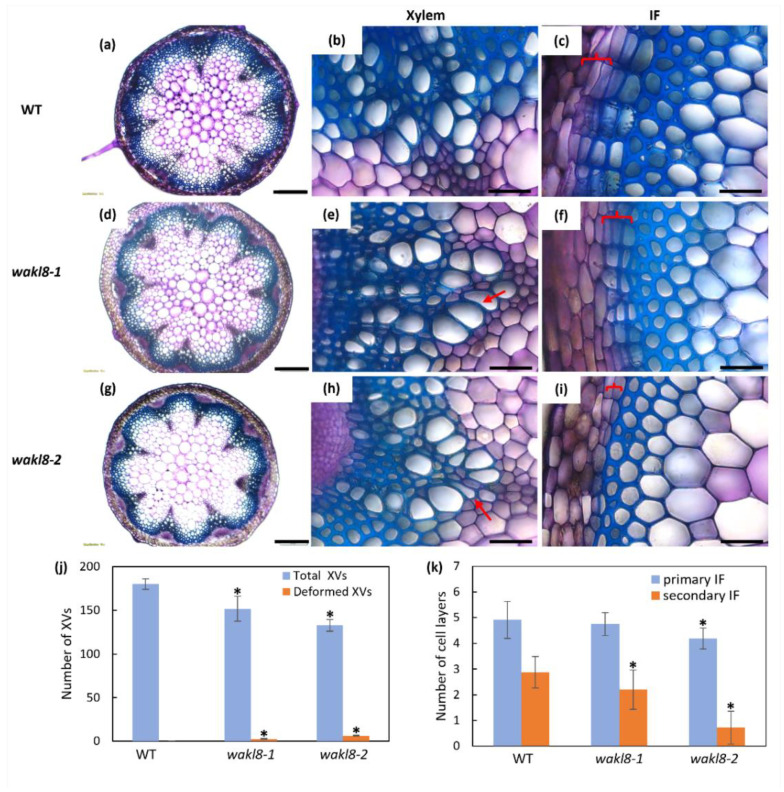
Histological analyses of transverse sections at 1 cm from the stem base of WT, *wakl8-1*, and *wakl8-2* plants. Fresh stems of WT (**a**–**c**), *wakl8-1* (**d**–**f**), and *wakl8-2* (**g**–**i**) plants at growth stage 6.5 [45] were sectioned and stained with Toluidine blue O. Thinner and slightly deformed xylem vessel (XV) walls could be observed in mutants compared with WT plants (red arrows in (**e**,**h**)). (**j**) Quantification of the number of XVs in stem transverse sections. (**k**) Quantification of primary and secondary interfascicular fiber (IF; red brackets) layers in stem transverse sections. Scale bar = 200 µm in (**a**,**d**,**g**), 20 µm in (**b**,**c**,**e**,**f**,**h**,**j**). Data shown as average ± SD acquired from three biological replicates. * Significant difference compared with WT plants, *p* < 0.05 using Student’s *t*-test.

To investigate if changes at the tissue level could explain the reduced stem length phenotype in *wakl8-2* mutants, histological analysis was performed of cellular organization in fresh stem sections taken at 1 cm above the base of plants at growth stage 6.5 [45]. Stem sections showed *wakl8-2* had slightly deformed and thinner XV walls compared with those of WT plants (Figure 4). The *wakl8-2* mutant also showed a reduced number of secondary IF layers compared with those of WT plants (Figure 4). Deformed XVs were also observed in *wakl8-1*, but the phenotype was less severe than in *wakl8-2* plants (Figure 4 compares d–f with g–i).

### 2.4. WAKL8 Regulates Stem SCW Synthesis

**Figure 5:**
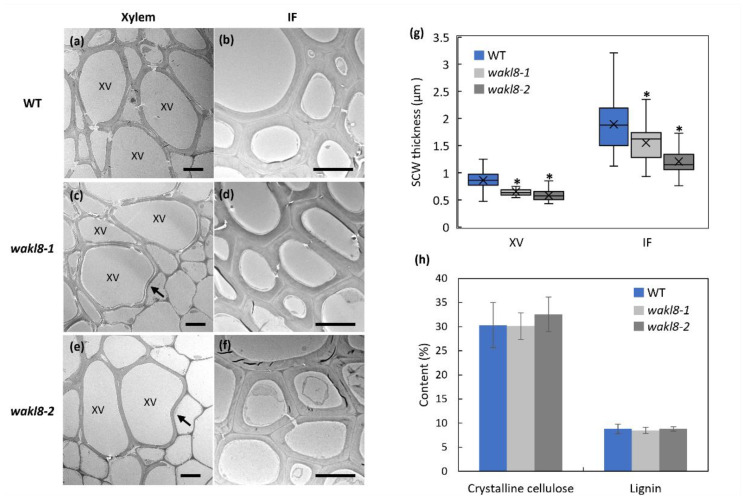
Transverse stem sections imaged by transmission electron microscopy (TEM) and measurement of stem crystalline cellulose and lignin contents. TEM imaging of xylem vessel (XV) and interfascicular fiber (IF) walls from WT (**a**,**b**), *wakl8-1* (**c**,**d**), and *wakl8-2* (**e**,**f**) plants at growth stage 6.5 [45]. Arrows indicate sites of collapsed XVs. Scale bar = 5 µm in (**a**–**f**). (**g**) Quantification of XV and IF wall thickness. (**h**) Measurement of crystalline cellulose and lignin contents in stems. Data shown as average ± SD acquired from three biological replicates. * Significant difference compared with WT plants, *p* < 0.05 using Student’s *t*-test.

Transmission-electron microscopy (TEM) of stems was used to investigate the changes in the XV cell morphology and wall thickness of mutants. Both *wakl8-1* and *wakl8-2* mutants showed slightly deformed XVs and thinner XV and IF walls compared with those of WT plants (Figure 5a–g). The phenotypes in *wakl8-2* plants were more severe than in *wakl8-1* plants (Figure 5a–g). Crystalline cellulose and lignin content of stems were also measured in mutants but showed no significant differences compared with those of WT plants at stage 6.5 (Figure 5h, Figures S2 and S3).

### 2.5. WAKL8 Has Alternative Spliced Transcripts

**Figure 6:**
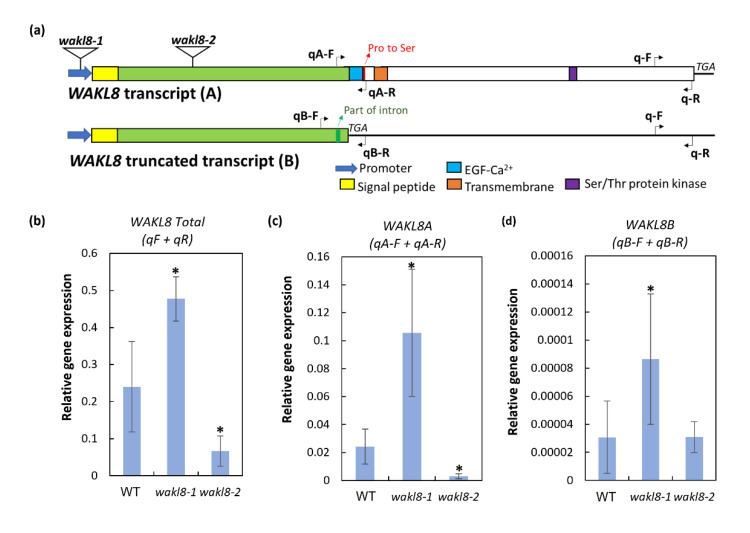
Schematic representation of *WAKL8* alternative spliced transcripts and Q-PCR analysis of transcript levels. (**a**) The *WAKL8A* transcript encodes a predicted protein with an N-terminal signal peptide (yellow), followed by an extracellular putative polysaccharide-interacting domain (green), EGF-Ca^2+^ domain (blue), transmembrane domain (orange), and cytoplasmic kinase domain (purple). The *WAKL8B* transcript has part of the first intron retained (red) and introduces a stop codon before the predicted EGF-Ca^2+^ domain. Positions are shown of T-DNA insertion in *WAKL8* to give *wakl8-1* and *wakl8-2* mutants and sites of primers used for Q-PCR analysis. (**b**) qPCR analyses of all *WAKL8* transcripts, (**c**) *WAKL8A* transcripts, and (**d**) *WAKL8B* transcripts in WT, *wakl8-1*, and *wakl8-2* mutant plants. Data shown as average ± SD acquired from 3 biological replicates. * Significant difference compared with WT plants, *p* < 0.05 using Student’s *t*-test.

Sequencing of *WAKL8* cDNA from Arabidopsis identified alternative spliced versions of *WAKL8* transcripts, which we named *WAKL8A* and *WAKL8B*. The *WAKL8A* transcript is a full-length transcript with all predicted protein domains (Figure 6a). The *WAKL8B* transcript showed part of the first intron expressed as an exon, which introduced a stop codon before the predicted EGF-Ca^2+^ domain and is predicted to encode a truncated WAKL8 protein that only contains the putative extracellular polysaccharide-interacting domain (Figure 6a and Figure S4). Q-PCR analysis using primers recognizing either all *WAKL8* transcripts, only *WAKL8A* transcripts, or only *WAKL8B* transcripts was used to compare the relative amounts of the different transcript versions in WT plants and mutants. In the WT plants, the *WAKL8A* transcripts were present at higher levels (approximately 1000-fold) than the *WAKL8B* transcripts (Figure 6b–d). In the *wakl8-1* mutants, *WAKL8A* transcripts were increased compared with those in the WT plants (Figure 6b–d). In the *wakl8-2* mutants, *WAKL8A* transcripts were significantly reduced compared with those in the WT plants, and *WAKL8B* transcripts were present at similar levels, suggesting the defects in SCW development in the *wakl8-2* mutants were unlikely to be regulated by *WAKL8B* transcripts (Figure 6b–d).

## 3. Discussion

Cell wall strengthening can be initiated in response to environmental (abiotic/biotic) stresses and is necessary for cells to acquire specific functions at defined spatiotemporal stages of normal growth and development. Whether plant SCW development is regulated by CWI mechanisms and what molecules and pathways are involved in this regulation remain unclear [3,46]. Arabidopsis SCW cellulose synthase mutant plants *cesa4*, *cesa7*, and *cesa8* have enhanced resistance to the soil-borne bacterium *P. cucumerina* and necrotrophic fungus *R. solanacearum*, indicating that CWI pathways can regulate the components of the SCW through CESAs [47]. Mechanical stresses, such as either bending or leaning, can induce reaction wood (RW) formation in either the lower (in gymnosperms, compression wood) or upper (in angiosperms, tension wood) sides of the stem, which display altered wall structure and chemical composition compared with nonstressed wood walls [48,49,50]. A few putative CWI genes have been identified that may play roles in regulating SCW sensing including *FLAs*, *COBRA-Like 4*, and homologues, *Vascular-Related Receptor-Like Kinase1* (*AtVRLK1*) [51,52,53,54,55]. Here, we show that WAKL8 functions in regulating SCW development and is another candidate CWI sensor.

Phenotypic analysis of *wakl8-2* mutant plants with decreased *WAKL8* transcript levels showed thinner XV and IF SCWs, suggesting a positive role of WAKL8 in regulating stem SCW development (Figure 3, Figure 4 and Figure 5). However, the *wakl8-1* mutant that had T-DNA insertion at the promoter region and increased *WAKL8* transcript levels also showed a mild phenotype of XV and IF SCW thickness (Figure 3, Figure 4 and Figure 5). A possible explanation for this unexpected result is that overexpression of *WAKL8* may interfere with or silence other WAKs/WAKLs, as there are at least 27 WAKs/WAKLs in Arabidopsis [15]. Generation of overexpression lines and analysis of other WAKs/WAKLs will be needed in future studies to clarify this inconsistency. The reductions in the numbers of XV and IF cells and decrease in SCW thickness, together with the lack of change in either cellulose or lignin contents (Figure 4 and Figure 5) suggest that WAKL8 plays a role as a regulator of SCW differentiation and development rather than specifically regulating either cellulose or lignin synthesis.

Mechanisms of how WAKL8 regulates stem SCW development remain to be explored, but a few hypotheses can be suggested. A role for pectin modifications regulating SCW development was previously identified, as shown by *POLYGALACTURONASE INVOLVED IN EXPANSION2* (*PGX2*) [56]. Overexpression of *PGX2* can increase stem SCW lignin content [56]. WAKs are known to initiate different signaling pathways based on interactions with different forms of pectin and pectin fragments. Arabidopsis WAK1 was shown to covalently interact with cell wall pectins and pectin fragments [19,57]. The interaction of WAK1 with pectin fragments can initiate defense responses to pathogens [19,57]. WAKL8 may act as a receptor of pectins ad pectin fragments, regulated by PGX2, to modulate SCW development. Future work is needed to confirm WAKL8-pectin binding, and crosses with *OE-PGX2* may reveal genetic interactions. The availability of sucrose to stems is a limiting step for stem SCW development, as shown by previous studies investigating the function of sucrose synthase (SUS) and invertase [58,59,60]. Yeast two-hybrid, fluorescence resonance energy transfer, and Phos-Tag assays showed WAKL8 can interact with phosphorylate SUC2 and positively regulate phloem loading, suggesting an alternative explanation for how WAKL8 can regulate stem SCW development [44]. The sucrose allocated to stems for XV and IF cell development may likely be limited in *wakl8* mutant plants because of the lower SUC2 activity, resulting in the defects in stem SCW thickening. However, measurements of sucrose level and SUC2 activity in *wakl8* mutant stems are required for testing this hypothesis. Further experiments are needed to investigate if WAKL8 can phosphorylate SUC2 (and other sucrose transporters) in stem tissues to regulate sucrose availability for stem SCW development, and to identify the putative protein interactors with WAKL8 and the downstream signaling pathways.

We also identified that *WAKL8* undergoes alternative splicing. Interestingly, a similar alternative splicing of WAKs was reported in maize *ZmWAK-RLK1* [27]. *ZmWAK-RLK1* was shown to regulate the hemi-biotrophic fungus *Exserohilum turcicum*, and an alternative spliced version, predicted to encode a truncated WAK, was identified but not suggested to contribute to pathogen resistance [27]. Although both ZmWAK-RLK1 and AtWAKL8 truncated version were suggested to be nonfunctional, we cannot exclude the possibility that these spliced version transcripts are important when plants are exposed to specific growth and/or stress conditions. As these truncated proteins still contain extracellular domains, they may competitively interact with pectins and pectin fragments to reduce the signaling strength under certain circumstances. It remains to be determined whether the alternative splicing of WAKs/WAKLs is conserved in different species and what mechanisms are involved in regulating the splicing of *WAK* transcripts.

## 4. Materials and Methods

### 4.1. Plant Materials

*Arabidopsis thaliana* wild0type (WT, Col-0), *wakl8-1* (SALK_029502C) and *wakl8-2* (WiscDsLox350H06) mutant plants were ordered from The Arabidopsis Biological Resource Center (ABRC, Ohio State University) and homozygous lines were identified by PCR genotyping. Primers used for *wakl8-1* genotyping were: LP (CGATATGGAGAAAGGGTCTCC), RP (CCCACACGAATTGTCATTTTC), and LBb1.3 (ATTTTGCCGATTTCGGAAC). Primers used for *wakl8-2* genotyping were: LP (TATGGGTCAAGGTCTTCGTTG), RP (GCCAGGTATGTAGGGATTTCC), and P745 (AACGTCCGCAATGTGTTATTAAGTTGTC). All plants were grown in controlled environment rooms under long-day 16 h light/8 h dark conditions at 22/18 °C.

### 4.2. Sequence Analysis and Protein Domain Prediction

Functional domains of WAKL8 (AT1G16260) were predicted online at UniProt (www.uniprot.org, accessed on 30 January 2018). *WAKL8* genomic DNA and mRNA sequences were downloaded from TAIR (www.arabidopsis.org, accessed on 30 January 2018). Benchling (https://benchling.com, accessed on 30 January 2018) was used for sequence alignment.

### 4.3. Q-PCR

Total RNA was extracted from stems at the same growth stages using an RNeasy kit (Qiagen). cDNA was synthesized with a SuperScript IV Reverse Transcriptase kit (Invitrogen). A QuantStudio 5 Real-Time System (Thermo Fisher, Waltham, MA, USA) was used for measuring transcript levels using the relative quantitation method [61] with PowerUp SYBR Green Master Mix (2X) Universal (#A25742, Thermofisher). Relative expression levels were normalized against *ACT2*. Three biological and three technical replicates were performed. The Q-PCR primers used for total *WAKL8* were: forward (GATCGCAATGCCGGAGTCTA) and reverse (TCACTGTGTCTTGTGAGGCA). For *WAKL8A*, the primers were: forward (GGCGGATGCCAAGACATT) and reverse (CAAGTTTTCTCACATCTATATGATCCG). For *WAKL8B*, they were: forward (GGATGCcaagtttggaattttt) and reverse (AGAGCTTAGAGTTCCACATCTATATGAT). For *ACT2*, they were: forward (ACATTGTGCTCAGTGGTGGA) and reverse (GAGATCCACATCTGCTGGAAT). The data are shown as average ± SD. Student’s *t*-test was used for significance analysis with *p* < 0.05.

### 4.4. Phenotyping Analysis

Arabidopsis plants at growth stage 6.1 [45] were used for measurements of leaf growth. Ten technical replicates from each of ten biological replicate plants were measured. Arabidopsis plants at growth stage 6.9 [45] were used for measurements of stem length. Six technical replicates from each of six biological replicate plants were measured. The data are shown as average ± SD. Student’s *t*-test was used for significance analysis with *p* < 0.05.

### 4.5. Histological Analyses

Fresh stems were hand-sectioned and stained with either Toluidine blue O, phloroglucinol-HCl, or Mäule stain to observe the cell morphology of stems according to methods outlined in Mitra and Loqué [62]. Images were acquired using an Olympus BX53 microscope under a bright field. At least three plants were used for quantifications. The data are shown as average ± SD. Student’s *t*-test was used for significance analysis with *p* < 0.05.

### 4.6. Transmission Electron Microscopy

Base stems were chemically fixed according to Wilson and Bacic [63]. Thin sections (~80 nm) were post-stained and imaged using a Jeol (Tokyo, Japan) 2100 EM equipped with a Gatan (Pleasanton, CA, USA) Orius SC 200 CCD camera. Three technical replicates from each of three biological replicate plants were imaged. Images of metaxylem vessels and primary IF cells (about 10 cells from each technical replicate) were used for quantification of cell wall thickness. The data are shown as average ± SD. Student’s *t*-test was used for significance analysis with *p* < 0.05.

### 4.7. Measurement of Crystalline Cellulose and Lignin Content

Arabidopsis whole stems of stage 6.5 were harvested for alcohol-insoluble residue (AIR) preparation according to Pettolino et al. [64]. The Updegraff method was used for crystalline cellulose content measurement [65]. Acetyl bromide method was used for lignin content measurement according to Chang et al. [66]. Three technical replicates from each of three biological replicate plants were measured. The data are shown as average ± SD. Student’s *t*-test was used for significance analysis with *p* < 0.05.

## 5. Conclusions

In this study, we revealed the role of WAKL8 in regulating Arabidopsis stem SCW development by phenotypically analyzing *wakl8-1* and *wakl8-2* mutant plants. We found alternative splicing of WAKL8 transcripts proposed to lead to a truncated extracellular variant. These findings extend our understanding of the biological functions of *WAKs/WAKLs* and bring new perspectives to help us understand the regulation of SCW development for editing better crops and trees in the future.

## Data Availability

All data are available in the main text or the Supporting Information.

## Supplementary Materials

The following supporting information can be downloaded at: https://www.mdpi.com/article/10.3390/plants11172297/s1: Figure S1: Expression profiles of *WAKL8* during development. Figure S2: Phloroglucinol-HCl staining of lignin in transverse sections 1 cm from the stem base of WT, *wakl8-1* and *wakl8-2* plants. Figure S3: Mäule staining of lignin in transverse sections 1 cm from the stem base of WT, *wakl8-1* and *wakl8-2* plants. Figure S4: Sequencing of alternative spliced versions of *WAKL8* transcripts.
