# Supplementary material for: WAKL8 Regulates Arabidopsis Stem Secondary Wall Development
Source: Plants (Basel). 2022 Sep 2;11(17):2297. doi: 10.3390/plants11172297 (PMC9460275; doi:10.3390/plants11172297)
Supplement: Supplementary file 1 [file plants-11-02297-s001.zip › plants-1836385-supplementary.pdf]

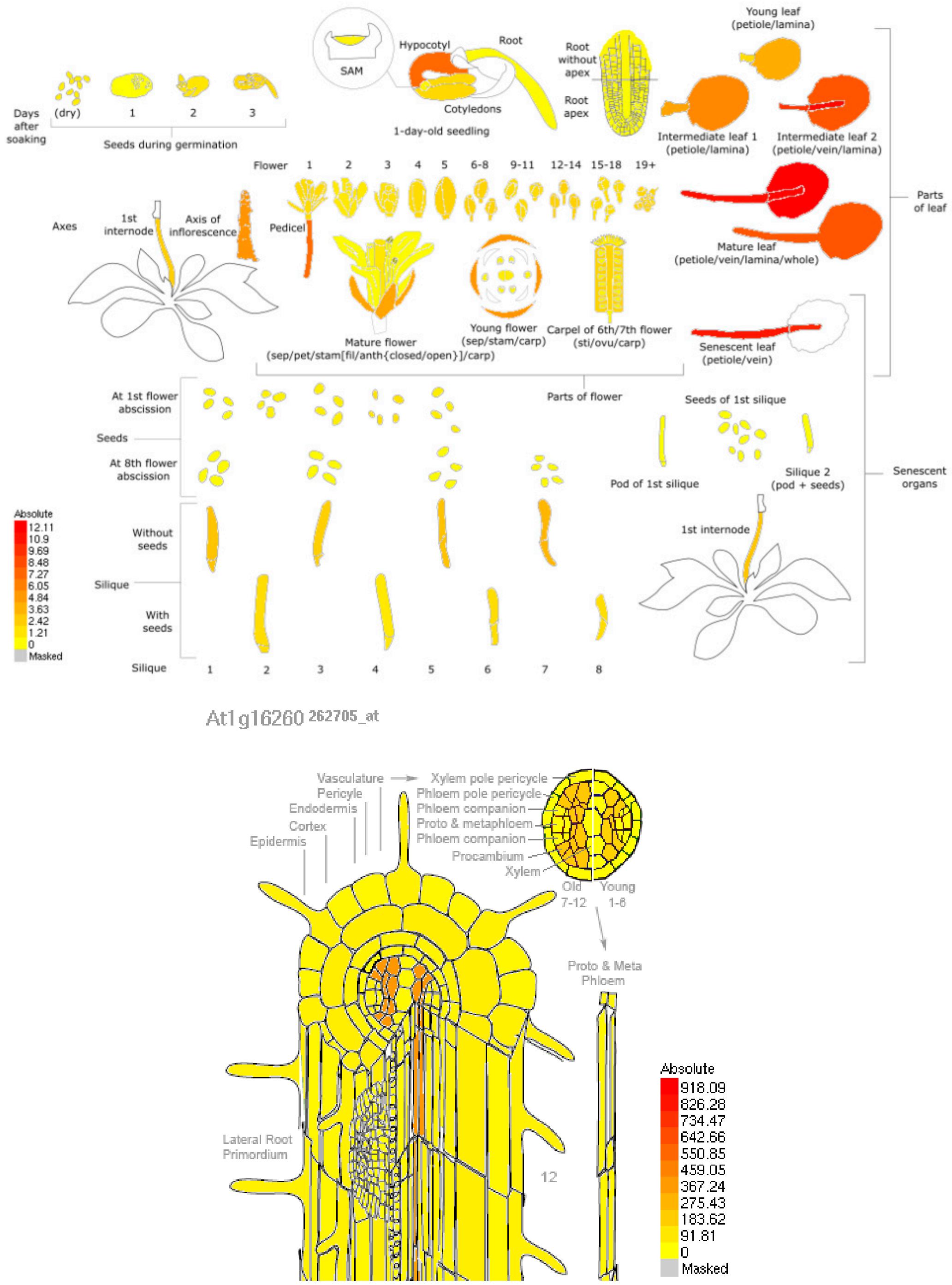

Figure S1. Expression profiles of *WAKL8* during development. Tissue level expression profile of *WAKL8* during Arabidopsis development visualized using eFP Browser [1,2]. The expression levels are represented as a colour gradient from yellow (lowest) to red (highest) and shows that *WAKL8* has slightly higher expression levels in stems and mature leaves than other tissues. In primary roots, *WAKL8* expression is highest in vascular cell types than other cells [1,3].

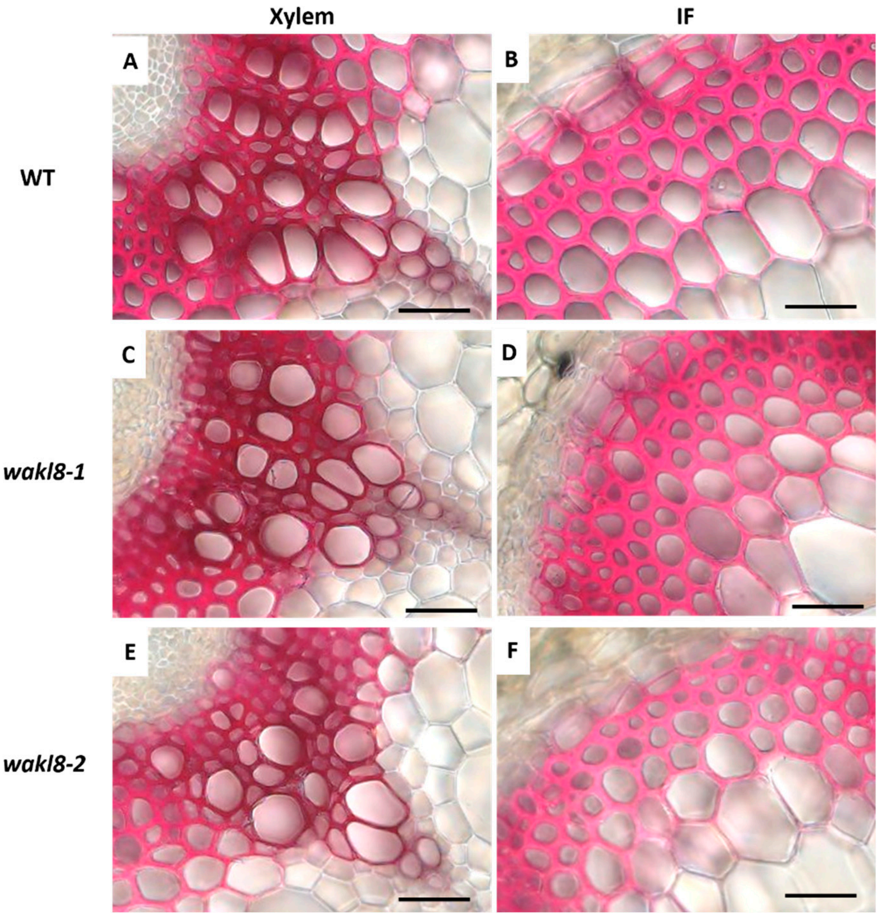

Figure S2. Phloroglucinol-HCl staining of lignin in transverse sections 1 cm from the stem base of WT, *wakl8-1* and *wakl8-2* plants. Fresh stems of WT (A-B), *wakl8-1* (C-D) and *wakl8-2* (E-F) plants at growth stage 6.5 were sectioned and stained with phloroglucinol-HCL. Scale bar = 20 µm.

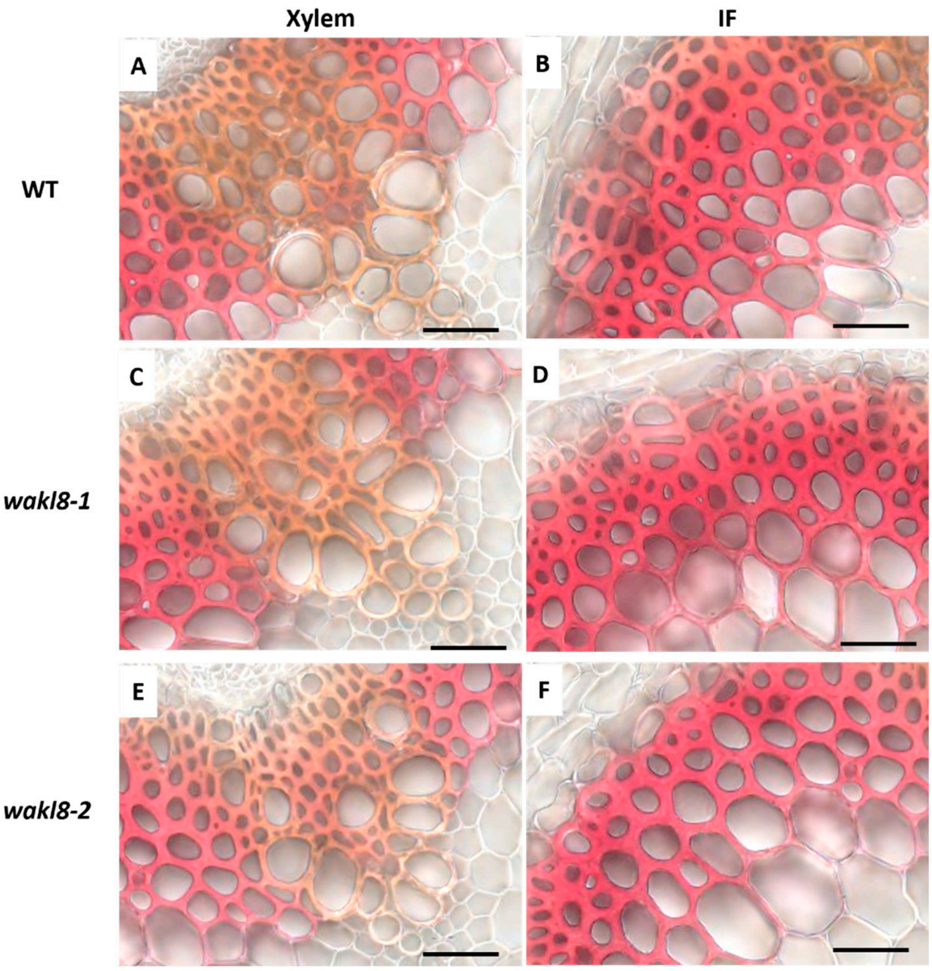

Figure S3. Mäule staining of lignin in transverse sections 1 cm from the stem base of WT, *wakl8-1* and *wakl8*-2 plants. Fresh stems of WT (**A**-**B**), *wakl8-1* (**C**-**D**) and *wakl8-2* (**E**-**F**) plants at growth stage 6.5 were sectioned and stained with Mäule. Scale bar = 20 μm.

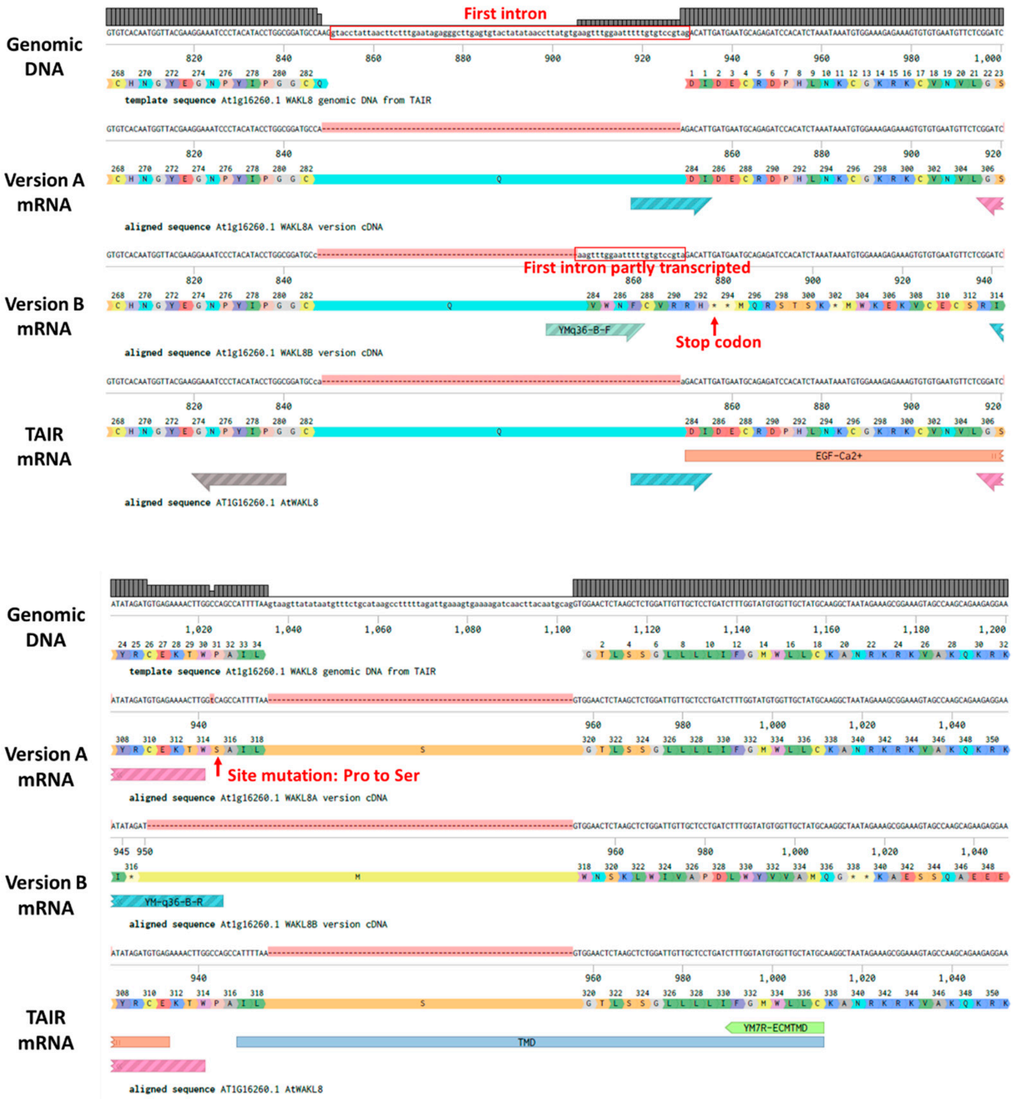

Figure S4. Sequencing of alternative spliced versions of *WAKL8* transcripts. PCR fragments cloned from wild-type (WT) plant stem cDNA were sequenced and aligned with WAKL8 genomic DNA and mRNA sequences downloaded from TAIR (https://www.arabidopsis.org/, accessed on 30 Jan 2018). *WAKL8A* version transcript had the same sequence as mRNA from TAIR but with a site mutation which caused an amino acid change from Pro to Ser before the predicted transmembrane domain (TMD). The *WAKL8B* version transcript had only a part of the first intron expressed, which caused a stop codon before the predicted EGF-Ca2+ region.

References

1. Winter, D.; Vinegar, B.; Nahal, H.; Ammar, R.; Wilson, G.V.; Provart, N.J. An "electronic fluorescent pictograph" browser for exploring and analyzing large-scale biological data sets. *PloS one* **2007**, *2*, e718.
2. Klepikova, A.V.; Kasianov, A.S.; Gerasimov, E.S.; Logacheva, M.D.; Penin, A.A. A high resolution map of the arabidopsis thaliana developmental transcriptome based on rna-seq profiling. *The Plant Journal* **2016**, *88*, 1058-1070.
3. Brady, S.M.; Orlando, D.A.; Lee, J.-Y.; Wang, J.Y.; Koch, J.; Dinneny, J.R.; Mace, D.; Ohler, U.; Benfey, P.N. A high-resolution root spatiotemporal map reveals dominant expression patterns. *Science* **2007**, *318*, 801-806.
